# Mutational signatures of colorectal cancers according to distinct computational workflows

**DOI:** 10.1093/bib/bbae249

**Published:** 2024-05-23

**Authors:** Paolo Battuello, Giorgio Corti, Alice Bartolini, Annalisa Lorenzato, Alberto Sogari, Mariangela Russo, Federica Di Nicolantonio, Alberto Bardelli, Giovanni Crisafulli

**Affiliations:** Department of Oncology, Molecular Biotechnology Center, University of Turin, Piazza Nizza 44, 10126, Turin, Italy; Genomics of Cancer and Targeted Therapies Unit, IFOM ETS, The AIRC Institute of Molecular Oncology, Via Adamello 16, 20139, Milan, Italy; Department of Oncology, Molecular Biotechnology Center, University of Turin, Piazza Nizza 44, 10126, Turin, Italy; Candiolo Cancer Institute, FPO - IRCCS, Strada Provinciale 142 - km 3.95, 10060, Candiolo, Turin, Italy; Candiolo Cancer Institute, FPO - IRCCS, Strada Provinciale 142 - km 3.95, 10060, Candiolo, Turin, Italy; Department of Oncology, Molecular Biotechnology Center, University of Turin, Piazza Nizza 44, 10126, Turin, Italy; Department of Oncology, Molecular Biotechnology Center, University of Turin, Piazza Nizza 44, 10126, Turin, Italy; Genomics of Cancer and Targeted Therapies Unit, IFOM ETS, The AIRC Institute of Molecular Oncology, Via Adamello 16, 20139, Milan, Italy; Department of Oncology, Molecular Biotechnology Center, University of Turin, Piazza Nizza 44, 10126, Turin, Italy; Genomics of Cancer and Targeted Therapies Unit, IFOM ETS, The AIRC Institute of Molecular Oncology, Via Adamello 16, 20139, Milan, Italy; Department of Oncology, Molecular Biotechnology Center, University of Turin, Piazza Nizza 44, 10126, Turin, Italy; Candiolo Cancer Institute, FPO - IRCCS, Strada Provinciale 142 - km 3.95, 10060, Candiolo, Turin, Italy; Department of Oncology, Molecular Biotechnology Center, University of Turin, Piazza Nizza 44, 10126, Turin, Italy; Genomics of Cancer and Targeted Therapies Unit, IFOM ETS, The AIRC Institute of Molecular Oncology, Via Adamello 16, 20139, Milan, Italy; Genomics of Cancer and Targeted Therapies Unit, IFOM ETS, The AIRC Institute of Molecular Oncology, Via Adamello 16, 20139, Milan, Italy

**Keywords:** genomics, computational biology, colorectal cancer, mutational signatures, bioinformatics, precision oncology, benchmarking

## Abstract

Tumor mutational signatures have gained prominence in cancer research, yet the lack of standardized methods hinders reproducibility and robustness. Leveraging colorectal cancer (CRC) as a model, we explored the influence of computational parameters on mutational signature analyses across 230 CRC cell lines and 152 CRC patients. Results were validated in three independent datasets: 483 endometrial cancer patients stratified by mismatch repair (MMR) status, 35 lung cancer patients by smoking status and 12 patient-derived organoids (PDOs) annotated for colibactin exposure. Assessing various bioinformatic tools, reference datasets and input data sizes including whole genome sequencing, whole exome sequencing and a pan-cancer gene panel, we demonstrated significant variability in the results. We report that the use of distinct algorithms and references led to statistically different results, highlighting how arbitrary choices may induce variability in the mutational signature contributions. Furthermore, we found a differential contribution of mutational signatures between coding and intergenic regions and defined the minimum number of somatic variants required for reliable mutational signature assignment. To facilitate the identification of the most suitable workflows, we developed Comparative Mutational Signature analysis on Coding and Extragenic Regions (CoMSCER), a bioinformatic tool which allows researchers to easily perform comparative mutational signature analysis by coupling the results from several tools and public reference datasets and to assess mutational signature contributions in coding and non-coding genomic regions. In conclusion, our study provides a comparative framework to elucidate the impact of distinct computational workflows on mutational signatures.

## Introduction

Genetic instability fuels tumor initiation and progression, and mutations represent the primary source of genetic variation. There is increasing evidence that a variety of factors can damage DNA and induce specific patterns of mutations in the genome, also known as mutational signatures [1–4]. Currently, there is no gold standard for mutational signature analysis. Since their initial discovery, more than thirty different bioinformatic tools [5] have been developed to extract *de novo* mutational signatures or to perform *fitting* analysis to estimate the prevalence of already characterized signatures in individual samples [1, 6]. This lack of standardization and the use of multiple analytical approaches may result in discrepancies. Furthermore, the number of signatures is continuously increasing: for example, the Catalogue Of Somatic Mutations In Cancer (COSMIC) [7] contained 30 single base substitution (SBS) mutational signatures as of March 2015 (v2) [1], while the latest version (v3.3) of July 2023 includes 79 SBS mutational signatures [8, 9]. Importantly, the inclusion of a low number of signatures in the analysis could lead to underestimation of active mutational processes in a tumor; while performing mutational signatures fitting on a large number of signatures could result in signal dilution and overfitting, as previously reported [10]. Furthermore, most of the reported signatures and the available tools for mutational signature profiling were designed to work with whole exome (WES) or whole genome (WGS) data [1, 8, 11]. Indeed, the extent to which next-generation sequencing (NGS) data from targeted gene panels (such as those used for clinical diagnosis or predictive purposes) can be exploited to reliably identify mutational signatures is largely unknown.

In this study, we assessed the impact of several arbitrary parameters on mutational signature analysis of human tumor samples. To this end, we focused on colorectal cancer (CRC) as a paradigmatic example of a common malignancy. According to their molecular profiles, most CRCs are classified as microsatellite stable (MSS) tumors, are characterized by chromosomal instability and are usually associated with a proficient mismatch repair machinery (MMRp). In contrast, microsatellite instable (MSI) tumors, representing a minor fraction of CRCs, generally display mismatch repair deficiency (MMRd) leading to a hypermutated phenotype [12, 13]. These molecular subtypes are associated with defined clinical features, such as anatomic site, treatment response and prognosis [14–19]. Notably, a small fraction of MSS-MMRp samples (1–2%) harbors mutations in the exonuclease domain of the DNA polymerase epsilon (*POLE*), resulting in a hypermutated phenotype paralleled by a good responsiveness to immune checkpoint blockade [16, 20]. As for other cancer types, the mutational signature profiles of CRC can be exploited for stratification purposes and to guide therapeutic decisions [21–26]. In this work, we evaluated how the choice of sequencing workflow, computational tools and mutational signature references affects signature analysis, both in terms of technical validity and the effectiveness of the resulting signatures in stratifying molecular subtypes of CRC. We exploited WES, WGS and the TruSight Oncology 500 targeted gene panel (TSO-500 from Illumina) [27] data, gathered by sequencing genomic DNA from CRC datasets, including 230 cell lines [28–31], 152 patients from The Cancer Genome Atlas (TCGA) [32] and validating our results in three independent datasets. Finally, to make our workflow accessible and usable, we developed CoMSCER (Comparative Mutational Signature analysis on Coding and Extragenic Regions), a bioinformatics tool capable of assessing the impact of multiple parameters on the robustness of the results to identify the most appropriate bioinformatic workflow.

## Methods

### Datasets

The preclinical dataset comprises a collection of CRC cell lines (Table S1) maintained as previously reported [29] and a publicly available clinical datasets from Genomic Data Commons (GDC) data portal repository under the TCGA project (TCGA-COAD). We validated our findings in independent datasets: a cohort of 483 endometrial cancer patients (TCGA-UCEC), 35 lung cancer patients (TCGA-LUAD and TCGA-LUSC) and 12 CRC PDOs [33].

### Genetic analysis

Maxwell RSC Blood DNA Kit was used for DNA extraction from cell lines and the preparation was performed following the manufacturer’s protocol. Starting from 400 ng of DNA from cell lines, WGS libraries were prepared using Nextera DNA Flex Library Preparation Kit according to the manufacturer’s protocol. For the preclinical dataset, fastq files were generated from Illumina Novaseq6000 and processed using the genomic analysis workflow as previously described [34, 35]. BWA-mem algorithm [36] was used to map reads to the human genome version 38 and PCR duplicates were removed using the RMDUP function in the SAMtools [37]. Mutations supported only by alteration in the first/last read position were filtered and strand bias correction was applied as previously described [34]. Starting from mutational files containing genetic alterations, only genetic alterations with fractional abundance  ≥ 10% were used for mutational signatures analysis. VCF files of samples in the clinical dataset (Table S1) and UCEC cohort were downloaded and filtered for the availability of clinical information concerning microsatellite and *POLE* status. ‘MAF’ files from the GDC lung cancer dataset were downloaded and filtered for genetic alterations with fractional abundance  ≥ 10% and clinical annotation concerning smoking status.

### Mutational signature analysis using genomic data of different size

Mutational signature fitting analysis was performed using R (version 4.1.2), the ‘MutationalPatterns’ version 3.4.0 package and COSMIC v3.2 as a signatures reference in three different datasets: 230 WES CRC samples, 63 WGS and 230 NGS targeted panel sequencing (Table S1). Concerning NGS targeted panel sequencing, TSO-500 from Illumina was chosen due to its large applicability in clinical settings and for its large size (523 genes). The TSO-500 dataset was created *in silico* from WES data upon mutations extraction based on the coding region of TSO-500 gene list. Mutational fitting was performed using ‘fit_to_signatures’ function with standard setting. Cosine similarity was assessed with the R function ‘cos_sim_matrix’ from MutationalPatterns package between the original mutational matrix (from SigProfilerMatrixGenerator) and the *reconstructed* matrix obtained using custom script publicly available on Github (https://github.com/pbattuello/MutationalSignatures). Cosine similarity distribution was plotted with ‘ggplot2’ R-package. Each mutational signature contribution was normalized ranging from 0 to 1, representing the percentage of mutations assigned to that specific mutational signature. As a percentage, this contribution resulted to be normalized also to the genomic size of the reference dataset: whether it was WGS, WES or TSO-500. Normalized contributions for the mutational signatures reported on COSMIC with *‘defective DNA mismatch repair’* as aetiology (SBS: 6–15–20-21-25-26-44) were taken into consideration and used for sample stratification. SBS10a-b were used instead for *POLE-*mutated sample stratification. ‘Flat signatures’ (SBS: 3–5–8-40-89) were defined, as previously proposed [10], as signatures in which the 96-mutational profile shows relatively even contribution of each trinucleotide context (<0.05%). ΔMMR was defined as the difference between the median contribution of MMRd-associated signatures between MSS-MMRp and MSI-MMRd samples. In the same manner, ΔPOLE was defined as the difference between the median contribution of POLE-associated signatures between *POLE* wild-type MSS-MMRp and *POLE*-mutated MSS-MMRp samples.

### Metanormal creation

The metanormal sample was created from WES data from 21 peripheral blood mononuclear cells (PBMCs) as previously reported [22]. For the metanormal generation, an equal number of reads were randomly taken from each of the samples and merged in a single fastq file. All the genetic analysis was repeated as described in the previous section using the metanormal sample as a matched normal.

### Systematic review of bioinformatic tools to analyse mutational signatures

We conducted a literature systematic review from the publicly available repository PubMed Central (PMC) database (https://www.ncbi.nlm.nih.gov/pmc/), using as the searching key *‘mutational signatures’* in the title or the abstract section. The literature search cut-off date was July 31^st^, 2023. From the SigProfiler suite SignatureProfilerAssignment was chosen as the most recent tool for mutational signature fitting analysis. SomaticSignatures tool was not available for fitting analysis. The five tools with most occurrences were included in the manuscript analysis unless the software was not available for use. Table S2 provides a comprehensive overview.

### Mutational signature analysis—algorithms comparison

Starting from mutational call files from WES, mutational matrices were generated using SigProfilerMatrixGenerator version 1.1.31. Then, mutational signature fitting was evaluated using five algorithms from current literature: ‘signature.tools.lib’ version 2.1.2, ‘SignatureAnalyzer’ version 0.0.8, ‘SigProfilerAssignment’ version 0.0.7, ‘deconstructSigs’ version 1.9.0 and ‘MutationalPatterns’ version 3.4.0. All algorithms were run in standard settings or following authors guidelines to minimize differences due to arbitrary settings and highlight differences due to different fitting approaches. Cosine similarity was calculated between the original mutational profile and the one reconstructed upon mutational signature fitting analysis using ‘cos_sin_matrix()’ function from ‘MutationalPatterns’ R-package. 230 cell lines from CRC cell bank and 132 samples (20 samples annotated as MSI-L were excluded from the analysis) from the clinical dataset were used in this analysis. Based on both mathematical and biological evaluations ‘MutationalPatterns’ was chosen as the tool most suited for CRC samples and therefore used in the other results and as part of CoMSCER analysis.

### Mutational signature analysis—reference evaluation

Mutational signature analysis was performed on WES data using ‘MutationalPatterns’ and COSMIC v2, v3.2 and CRC-specific as reference dataset [8].

### Inferring a minimum number of mutations

We performed random sampling by 5% using the ‘*shuf’* function version 8.30 from ‘GNU coreutils’ for each sample of the two datasets (Table S1). 19 subgroups of mutations (from 5% to 95% using 5% interval) were identified for each sample; five different replicates were created for each subset and mutational signatures fitting analysis was performed for each subset as described in the previous methods section. Cosine similarity was calculated for each sample as reported and the median value was plotted using R-package ggplot2 version 3.3.5.

### Statistical analysis

Statistical analysis was performed using R version 4.1.2. The individual statistical tests are specified in the results section and figure legends. Wilcoxon rank sum test was performed using R function ‘*wilcox.test’* and ‘*’, ‘**’, ‘***’, ‘****’ footnotes were used to mark significance level, respectively *P* < 0.05, *P* < 0.01, *P* < 0.001, *P* < 0.0001.

### Data/code availability

All the code and data necessary to reproduce the study are available on GitHub repository (https://github.com/pbattuello/MutationalSignatures). NGS data are available at the European Bioinformatics Institute in the European Nucleotide Archive (ENA) with PRJEB33045, PRJEB33640, PRJEB57691 and PRJEB61897 accession codes. Cell lines were selected based on the availability of genomic data from NGS (Table S1). Compared to the datasets we reported previously [28–30], additional cell lines WGS were included in the current cohort. Idea tool for mutational calling pipeline is available at (https://bitbucket.org/irccit/idea/src/master/) [34]. CoMSCER is available at https://github.com/pbattuello/CoMSCER.

## Results

### Identification of the main variables for mutational signature analysis and workflow design

**Figure 1:**
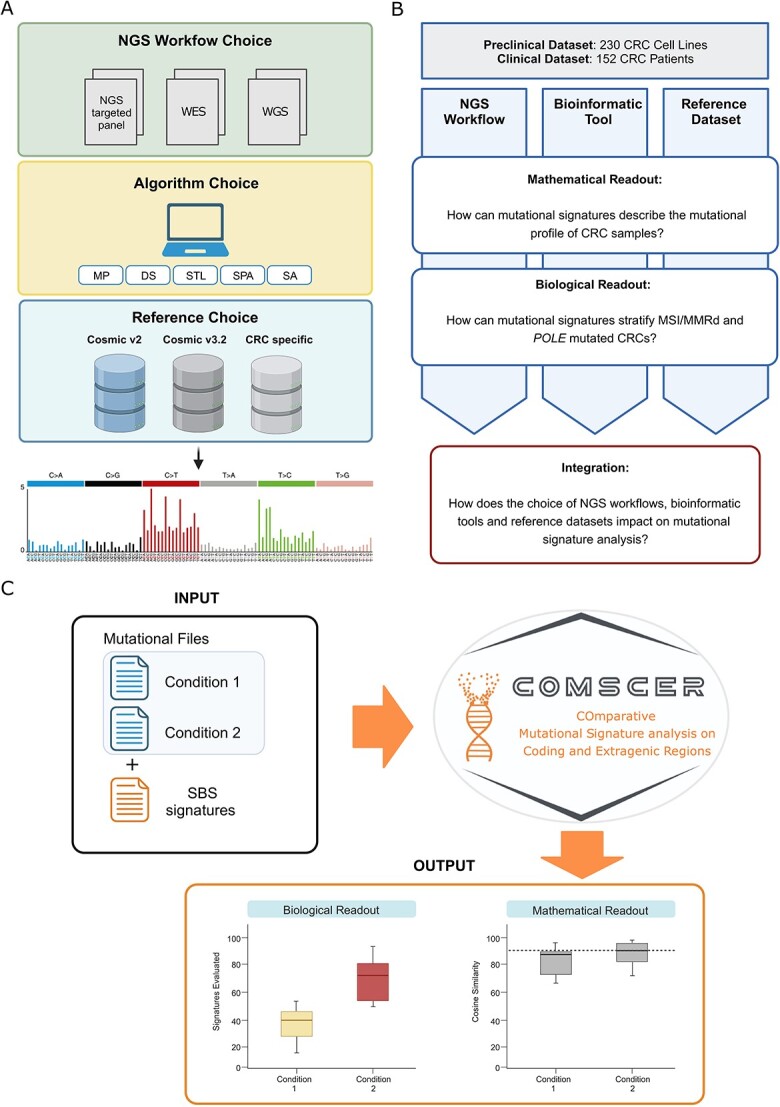
*Graphical representation of the experimental workflow used to determine key variables for mutational signature analysis.* (A) A prototypic bioinformatic pipeline for mutational signature profiling is graphically represented. (B) Experimental workflow of the study. (C) The CoMSCER workflow is graphically represented. *NGS, Next Generation Sequencing; WES, Whole Exome Sequencing; WGS, Whole Genome Sequencing; VCF, Variant Call Format; COSMIC, Catalogue Of Somatic Mutations In Cancer; CRC, Colorectal Cancer; MP, MutationalPatterns; DS, deconstructSigs; STL, signature-tools.lib; SPA, SigProfilerAssignment; SA, signatureanalyzer*.

As a starting point, we reasoned that defining the key steps of a ‘standard’ bioinformatic pipeline would allow identifying the main variables of the analysis. We identified three key parameters: the NGS workflow, the bioinformatic tool for the signature fitting analysis and the reference catalogue of mutational signatures (Fig. 1A). For the first parameter, we performed mutational signature analysis on three different types of NGS data: the TSO-500 pan cancer panel, WES and WGS data (Table S1). For the second variable, we performed a systematic review of the literature which resulted in determining the five most commonly used bioinformatic software for mutational signature profiling (Table S2) including: MutationalPatterns [38], deconstructSigs [39], signature.tools.lib [40], SigProfilerAssignment [41] and SignatureAnalyzer [8]. Finally, we evaluated how the reference mutational signatures could influence the overall results considering two versions of the COSMIC mutational signatures catalogue (v2 and v3.2) and a tissue-specific signature reference [1, 7, 8] (Fig. 1A). Next, we designed a computational workflow to evaluate how each parameter could influence the overall signature analysis, using two different readouts (Fig. 1B). The first is a mathematical readout, assessing how fitted mutational signatures recapitulate the mutational landscape of individual samples. This is measured by calculating the cosine similarity between the mutational profile of each sample and the profile reconstructed using the fitted mutational signatures, considering 0.9 as the cosine similarity threshold as previously reported (‘not assigned’ < 0.9) [42]. The second is a biological readout, defined as the ability of the fitted mutational signatures to properly stratify two biologically relevant CRC subtypes: MSI-MMRd and *POLE*-mutated hypermutant CRCs. To assess the ability of the fitted signatures to discriminate MMRp from MMRd tumors, we calculated the median ΔMMR parameter, defined as the difference of the median contribution of fitted MMR deficiency signatures between MSI-MMRd and MSS-MMRp samples: the higher the value, the higher the ability of fitted signatures to discriminate MSI-MMRd and MSS-MMRp samples. Similarly, we calculated the median ΔPOLE parameter, defined as the difference of the median contribution of fitted *POLE* signatures between *POLE*-mutated and *POLE* wild-type samples, to assess the ability of signatures to classify CRC based on *POLE* mutational status (Fig. 1B).

### Comparative mutational signature analysis on coding and extragenic regions

We considered that a bioinformatic tool which comprehensively and systematically performs the above-mentioned analyses in multiple datasets originating from distinct tumor types is not available. To address this knowledge gap, we developed CoMSCER, a freely available bioinformatic tool. By specifying the SBS mutational signatures of interest (e.g., MMRd, treatment induced) and two given conditions (e.g., MMRp versus MMRd, pre versus post treatment), CoMSCER evaluates the mathematical and biological readouts from multiple bioinformatic tools, reference datasets and differential signature contribution between coding and non-coding regions for the identification of the most suited bioinformatic workflow (Fig. 1C, https://github.com/pbattuello/CoMSCER).

### Datasets for comprehensive mutational signature analyses

**Figure 2:**
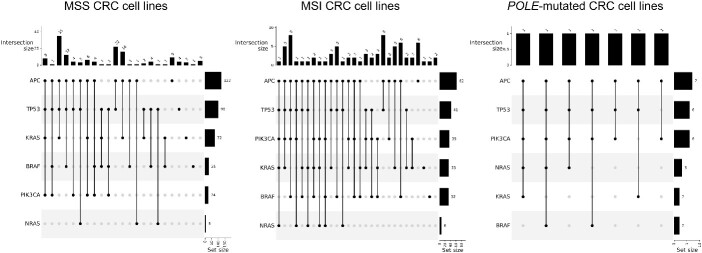
*Molecular features of the preclinical dataset*. Upset plots of the preclinical dataset reporting the genetic features of the CRC cell lines cohort. Cell lines are divided by genetic subtype: MSS-MMRp, MSI-MMRd and *POLE*-mutated. *MSS, Microsatellite Stable; MSI, Microsatellite Instable.*

We focused our analysis primarily on a preclinical dataset of CRC cell lines. This dataset comprises 187 CRC cell lines previously genotyped by our research group [28–31]. Additional genomic data from CRC cell lines were incorporated through this study, expanding the dataset to a total of 230 genomically annotated cell lines encompassing all the main CRC subtypes such as MSS-MMRp (145/230, 63%), MSI-MMRd (78/230, 34%) and POLE-mutated samples (7/230, 3%). Detailed genetic characteristics of the dataset are summarized in Fig. 2 and Table S1.

### Identification of mutational signatures using a targeted sequencing panel

**Figure 3:**
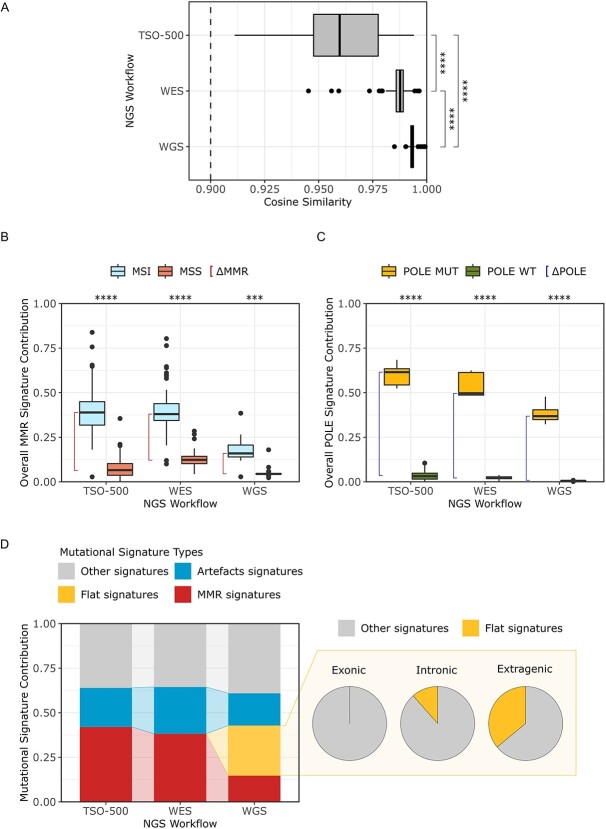
*Impact of the Next Generation Sequencing Workflows on Mutational Signature Profiling.* (A) Distribution of cosine similarity values in the preclinical CRC dataset, using WGS, WES and the TSO-500 pan-cancer panel. (B) Overall contribution of MMR associated mutational signatures in MSI-MMRd and MSS-MMRp CRC preclinical samples. (C) Overall contribution of *POLE* mutation-associated signatures in *POLE*-mutated and *POLE* wild-type CRC preclinical samples. (D) On the left, median contribution of artefact-driven, MMRd-associated and *‘flat signatures’* in the MSI-MMRd CRC cell lines. On the right, pie charts of the contribution of *‘flat signatures’* in exonic, intronic and extragenic regions from WGS data. *NGS, Next Generation Sequencing; MSS, Microsatellite Stable; MSI, Microsatellite Instable; WT, Wild-Type; MUT, Mutated; MMR, Mismatch Repair; WES, Whole Exome Sequencing; WGS, Whole Genome Sequencing.*

To assess the impact of NGS workflow choice, we investigated how different sequencing data, namely WGS, WES and the targeted pan-cancer panel TSO-500 (performed on the same samples) affect mutational signatures (Table S1). When considering the mathematical readout, cosine similarity reached the reliability threshold of 0.9 with all three NGS types of data, supporting the technical feasibility of the analysis spanning from WGS to gene-targeted panels. However, the three outcomes were significantly different when compared using the Wilcoxon rank test (Fig. 3A).

Concerning the biological readout (definition of CRC molecular subtypes), the median ΔMMR was >0 both using TSO-500, WES and WGS (Fig. 3B), suggesting that the use of all three data types allows significant stratification of MSS-MMRp from MSI-MMRd CRCs (Wilcoxon rank test, WES, TSO-500 and WGS, respectively *P* < 2.2e-16, *P* < 2.2e-16 and *P* = 1.03e-04, Fig. 3B). A similar scenario was observed when considering sample stratification based on *POLE* mutational status assessed by median ΔPOLE parameter (Fig. 3C).

However, when the median ΔMMR was higher than 0 with all three sequencing workflows, the value was unexpectedly lower using WGS data (0.11 WGS < 0.26 WES < 0.32 TSO-500). We hypothesized that this could be due to the dilution of the MMR signature signal with larger genomic sources such as WGS. Therefore, we investigated the contribution of different classes of mutational signatures in our databases of MSI-MMRd CRC cell lines. Specifically, we considered: MMRd related signatures, a specific signatures often referred to as *‘flat signatures’* [10]*,* artefact-associated signatures and mutational signatures associated with unrelated biological processes. This analysis showed an increased signal for *‘flat signatures’* in WGS data (Fig. 3D), thus suggesting a possible explanation for the previously observed signal dilution. Additionally, to elucidate the possible source of the increase of *‘flat signatures’* signal, we asked whether distinct genomic regions may contribute differently to mutational signature contribution. We performed mutational signature analysis considering mutations derived from the exonic, intronic and extragenic regions extracted from WGS. The analysis showed lack of *‘flat signatures’* in the exonic regions, confirming the results from WES, while intronic and extragenic regions exhibited an increment of the *‘flat signatures’* contribution of 11,6% and 36%, respectively (Fig. 3D). To further support and extend these results, we evaluated the median ΔMMR in each specific genomic region. As highlighted in Fig. S1, median ΔMMR between MSI-MMRd and MSS-MMRp CRC cell lines of the extracted exonic regions aligns closely with that observed from WES data.

Overall, these results indicate that mutational signature analysis may be feasible not only using WES and WGS data but also large pan-cancer NGS panels such as the TSO-500. Notably, increasing the genomic size evaluated in the analysis was only partially helpful in improving signature accuracy.

### Impact of computational algorithms on mutational signature analysis in CRC

**Figure 4:**
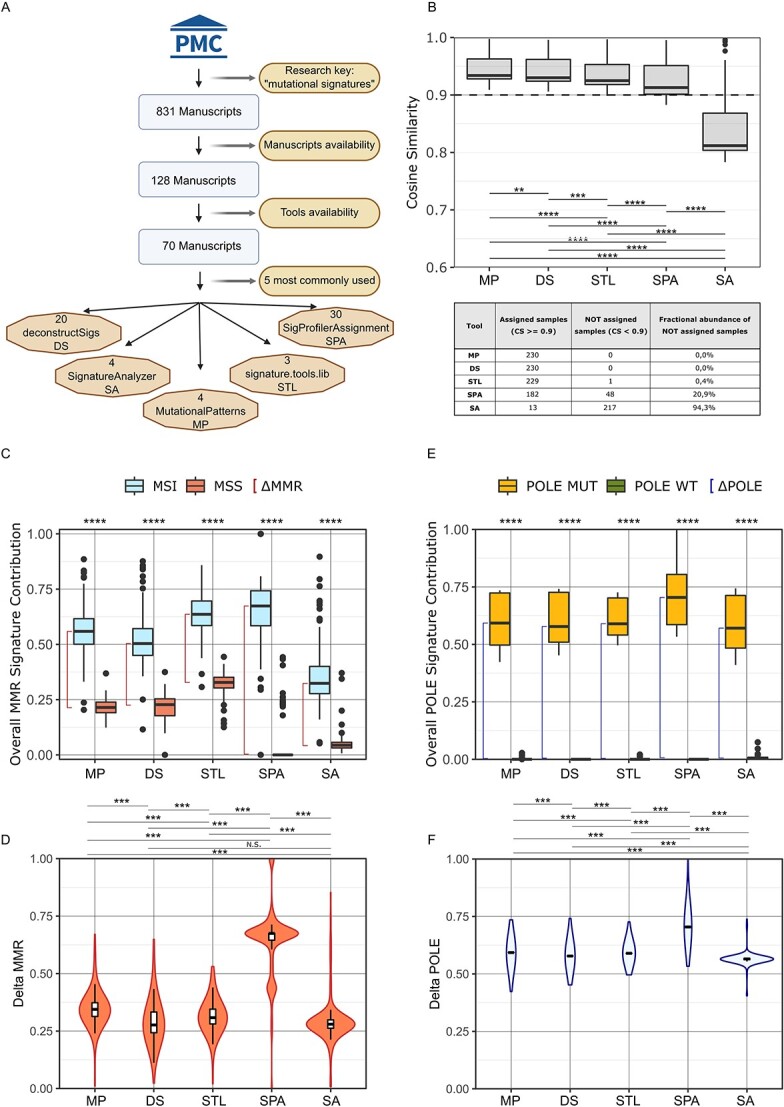
*Impact of algorithm choice on mutational signatures analysis in the preclinical dataset.* (A) Graphical representation of the systematic review utilized to identify the 5 most used tools. (B) Distribution of cosine similarity values obtained with *MutationalPatterns, deconstructSigs, signature-tools.lib, SigProfilerAssignment and signatureanalyzer* in the preclinical dataset. (C) Overall contribution of MMRd-associated signatures in MSI-MMRd and MSS-MMRp cell lines according to the indicated algorithms. (D) Distribution of ΔMMR values according to the indicated algorithms (E) Overall contribution of *POLE* associated mutational signatures according to the indicated algorithms. (F) Distribution of ΔPOLE values according to the indicated algorithms. *PMC, PubMed Central; MP, MutationalPatterns; DS, deconstructSigs; STL, signature-tools.lib; SPA, SigProfilerAssignment; SA, signatureanalyzer; n.s., not significant*.

We conducted a literature systematic review on PMC using as search key ‘mutational signatures’. From the initial 831 entries, 128 manuscripts were available for download and are listed Table S2. From this pool, we identified 70 papers that referenced algorithms for fitting mutational signatures that were both available and installable. From this list, we selected the top five most referenced tools (Fig. 4A). We next performed mutational signature fitting using the five bioinformatic tools identified: *MutationalPatterns* (MP)*, deconstructSigs* (DS)*, signature-tools.lib* (STL)*, SigProfilerAssignment* (SPA) *and SignatureAnalyzer* (SA) on the CRC preclinical and clinical datasets [32].

In the preclinical dataset, four out of five tools reached a median cosine similarity of 0.9. Differences in cosine similarity distribution among the five software were statistically significant (Fig. 4B, upper panel). We highlighted that, among the five tools evaluated, SPA and SA did not allow the assignment of more than 20% of the samples (1/230, 0.4% with STL, 48/230, 20.9% with SPA and 217/230, 94.3% with SA). Notably, only MP and DS allowed mutational signature fitting for all 230 samples (Fig. 4B, lower panel). Results from the clinical dataset were comparable: four out of five software reached a median value of cosine similarity above the technical reliability threshold, with only limited samples not reaching the threshold. Similar to what we observed in the preclinical dataset, cosine similarity distributions were significantly different (Fig. S2A, upper panel). Also in this case, multiple samples were not assigned by different tools: 1/152, 0.66% with MP, 10/152, 6.6% with DS, 12/152, 7.9% with STL, 25/152, 16.4% with SPA 116/152, 76.3% with SA (Fig. S2A, lower panel). Of note, the trend between the median value of cosine similarity among the five different algorithms was maintained across the preclinical and clinical datasets.

Next, we evaluated the ability of each bioinformatic tool to correctly stratify MSS-MMRp and MSI-MMRd tumors (Fig. 4C). In the preclinical dataset, the MMR deficiency signature contribution between MSS-MMRp and MSI-MMRd samples was significantly different for all five software (Wilcoxon rank sum test, *P* < 2e-16). Nevertheless, SPA proved to have the highest MMRd signature fitting ability as indicated by the highest median MMR signature contribution obtained in MSI-MMRd samples with this tool (Fig. 4C). Furthermore, to properly compare the tools performance in discriminating MSS-MMRp and MSI-MMRd tumors, we analysed the ΔMMR distribution between MSI-MMRd and MSS-MMRp samples. This analysis highlighted significant differences between the contribution of MMR signatures in MSS-MMRp and MSI-MMRd using different algorithms. Notably, SPA provided the highest median separation between the two subtypes (MP = 0.34, DS = 0.28, STL = 0.31, SPA = 0.67, SA = 0.28, Fig. 4D).

Finally, to evaluate how mutational signatures stratify CRC *POLE*-mutated phenotype, we considered the *POLE*-related signature SBS10 (Fig. 4E) and ΔPOLE distribution (Fig. 4F). In the preclinical datasets, a significant difference was reported for all five algorithms. Considering *POLE* related signatures contribution, SPA showed again the highest values (MP = 0.59, DS = 0.58, STL = 0.59, SPA = 0.7, SA = 0.57).

The analysis of the clinical dataset revealed similar results for both MSI-MMRd/MSS-MMRp and MSS *POLE-*mutated/MSS *POLE* wild-type stratification. Genetic stratification of MSI-MMRd and MSS-MMRp patients was statistically significant for all algorithms (ΔMMR MP = 0.61, DS = 0.67, STL = 0.77, SPA = 1, SA = 0.26, Fig. S2B–C) and concordant results were also obtained for *POLE* stratification (ΔPOLE clinical dataset: MP = 0.59, DS = 0.68, STL = 0.77, SPA = 0.71, SA = 0.85, Fig. S2D–E).

To assess if software dependent differences persisted across different tumor types, we extended the analysis to an independent dataset comprising samples from endometrial tumors of 483 patients. These additional analyses confirmed the consistency of our results (Table S3). These findings highlighted that, depending on the tool of choice, more than 30% of samples remain ‘not assigned’ (Table S3). Finally, an additional validation of these divergent software performances was conducted using a different biological readout and tumor type. We focused on a dataset of lung tumors, classifying them based on smoking status, the outcome of these analyses further confirmed the results obtained in the CRC dataset (Table S3).

### Impact of different reference mutational signatures on CRC genetic characterization

Following the same strategy as above, we assessed how the mutational signature reference impacts mutational signature fitting and CRC molecular stratification. We selected three distinct references: COSMIC v2 (C2), COSMIC v3.2 (C3) [1, 8] and a CRC tissue-specific signature catalogue (TS) [40], each containing a different number of mutational signatures (30 in C2, 72 in C3 and 26 in TS).

**Figure 5:**
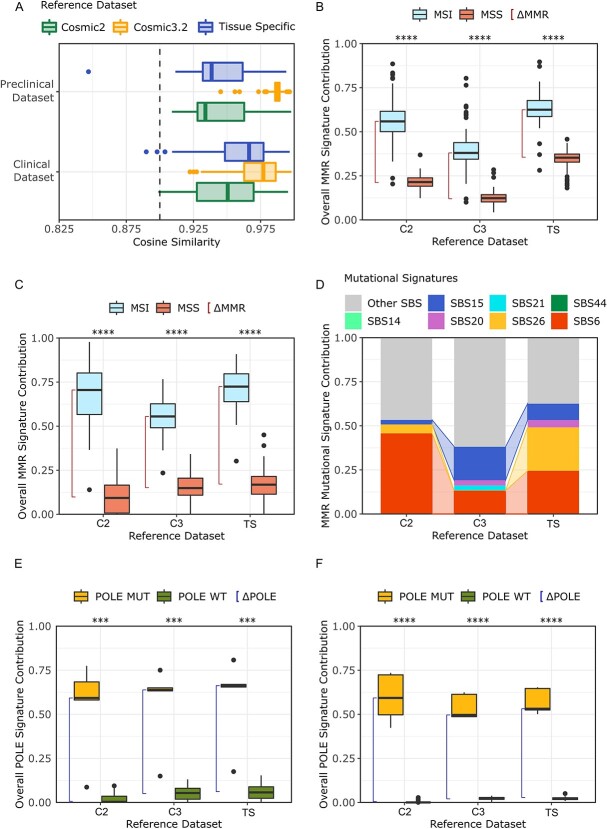
*Impact of the reference on mutational signatures analysis.* (A) Distribution of cosine similarity using three different signatures references in the clinical and preclinical datasets. (B) Contribution of MMRd-associated signatures in the CRC cell line dataset using three different signature references. (C) Contribution of MMRd-associated signatures in the clinical dataset using three different references. (D) Normalized contribution of single MMR-associated signatures in the MSI-MMRd subset of the CRC cell line dataset. (E) Contribution of *POLE*-associated signatures in the clinical dataset using three different references; Red line represents ΔPOLE (F) Contribution of POLE associated signatures in the preclinical dataset using three different references; Red line represents ΔPOLE. *COSMIC, Catalogue Of Somatic Mutations In Cancer; SBS, Single Base Substitution; MSI, Microsatellite Instable; MSS, Microsatellite Stable; C2, Cosmic v2; C3 Cosmic v3.2; TS, Tissue Specific.*

Cosine similarity analysis showed values above the reliability threshold with all references, with higher values corresponding to larger references. Differences were statistically significant in both the preclinical (Wilcoxon rank sum test, C2vsTS, C2vsC3, C3vsTS, respectively *P* = 1.2e-13, *P* < 2.2e-16, *P* < 2.2e-16) and the clinical dataset (Fig. 5A).

With respect to the ability to define CRC molecular subsets, all references obtained a significant ΔMMR, thus allowing proper identification of MSS-MMRp and MSI-MMRd (Wilcoxon rank sum test, C2vsTS, C2vsC3, C3vsTS, *P* < 2e-16, Fig. 5B–C) even if minor differences were present (preclinical dataset: ΔMMR C2 = 0.34, ΔMMR C3 = 0.26, ΔMMR TS = 0.27; clinical dataset: ΔMMR C2 = 0.61, ΔMMR C3 = 0.41, ΔMMR TS = 0.56). To further investigate if the reference choice could alter the contribution of a distinct mutational signature associated with MMR deficiency, we compared the contribution of each MMR deficiency signature in the MSI-MMRd cohort of the preclinical dataset. Of note, a certain variability was present, particularly in case of SBS6 (46% in C2, 13% in C3 and 24% in TS), SBS15 (3% in C2, 19% in C3 and 9% in TS) and SBS26 (5% in C2, 0% in C3 and 25% in TS, Fig. 5D). Comparable results were obtained when we evaluated the contribution of specific MMRd signatures in the clinical dataset (Fig. S3A).

We further performed the analysis in an independent dataset of 167 endometrial cancers annotated for MSI-MMRd status. Even in this scenario, the use of different mutational signature references led to changes in the contribution of individual signatures: SBS6 decreased from 73% in C2 to 43% and 32% respectively in C3 and in the TS references; while SBS21 emerged only in C3, SBS26 and SBS44 emerged only using the TS reference (Fig. S3B).

Finally, we considered *POLE* genetic stratification: in both CRC datasets, all references led to effective discrimination of *POLE-*mutated from *POLE* wild-type CRCs (Wilcoxon rank sum test, C2vsTS, C2vsC3, C3vsTS, *P* < 2e-16) (preclinical dataset: respectively ΔPOLE = 0.59, ΔPOLE = 0.47, ΔPOLE = 0.51; clinical dataset: ΔPOLE = 0.59, ΔPOLE = 0.59, ΔPOLE = 0.61, Fig. 5E–F).

In summary, the size of the mutational signature reference can impact the molecular stratification of CRC samples, specifically when distinct mutational signatures are considered.

### Inferring a minimum number of mutations for reliable mutational signature analysis

**Figure 6:**
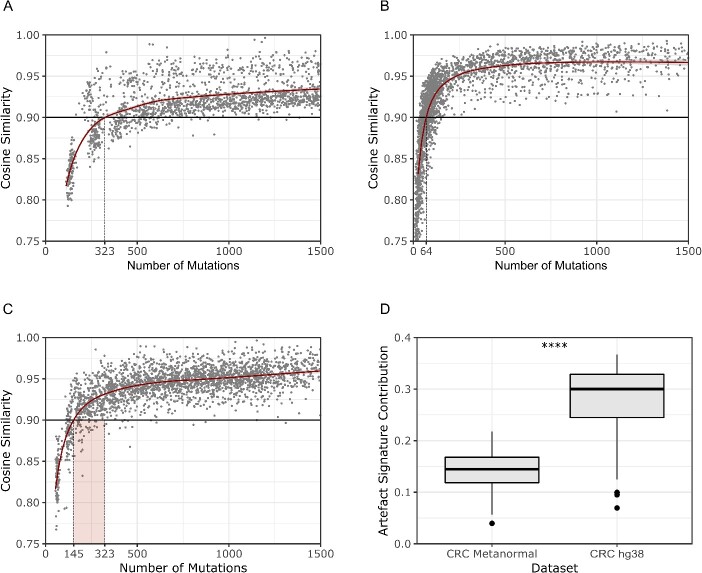
*Inferring the minimum number of mutations required to perform mutational signatures analysis.* (A) Sampling experiment on the CRC cell line dataset showing that at least 323 mutations are required to reach the threshold of cosine similarity for proper analysis. (B) Sampling experiment on the clinical dataset showing that at least 64 variants are required to reach the threshold of cosine similarity for performing the analysis. (C) Sampling experiment in the CRC cell line dataset using a metanormal as matched normal, showing that 145 mutations are needed for reaching the threshold (as compared to 323 in the absence of the metanormal). (D) Decrease in the contribution of mutational signatures associated with artefacts in the CRC cell line dataset using a metanormal. *CRC, Colorectal Cancer; hg38, human genome version 38.*

The discrepancy observed in the WGS based analysis between its high technical reliability (Fig. 3A) and its lower effectiveness to stratify CRC samples when compared to smaller size NGS workflows (Fig. 3B–C) was unexpected. To further investigate this aspect, we inferred the minimum number of mutations required to achieve a reliable mutational signature fitting. In particular, using both the CRC cell lines and the clinical dataset, we performed random sampling from 5 to 95% of all the mutations in each sample. Next, to establish the minimum number of mutations required to obtain technically robust results, we evaluated the cosine similarity. In the CRC preclinical dataset, 323 mutations were needed to reach the cosine similarity reliability threshold (Fig. 6A). The value plunged to 64 mutations for the clinical dataset (Fig. 6B). We reasoned that this discrepancy could be related to the specific features of the two datasets. Indeed, whilst the clinical datasets contain CRC versus matched healthy tissue, the preclinical CRC dataset lacks a non-malignant control line. To understand the impact of this discrepancy, we investigated to what extent the use of a matched normal affect mutational signature calling by decreasing the background originating from germinal variants and sequencing artefacts. For this purpose, we established a ‘metanormal’ obtained from 21 PBMCs of CRC patients and performed the mutational calling of the entire CRC cell line dataset using the metanormal as a normal sample [22]. In this instance, the number of mutations required to reach the cosine similarity threshold decreased from 323 to 145 (−55%, Fig. 6C).

Finally, we investigated how the use of a metanormal could impact the occurrence of mutational signatures associated with artefacts: the overall signal of artefact SBS signatures dropped from 0.30 to 0.15, thus confirming the effectiveness of this approach (Fig. 6D). To facilitate the reproducibility of this approach, the list of the genomic position for filtering was included in CoMSCER.

## Discussion and conclusion

Assessing the mutational signatures that characterize cancer genomes has biological and clinical implications, as reported in melanoma breast and colorectal tumors [22, 25, 43]. When our group started exploiting mutational signatures to interrogate clinical response to a new therapeutic approach in CRC patients, we realized that standardized methods to perform mutational signature analysis were not available causing a lack of reproducibility and robustness of the results [22] (clinical trial:NCT03519412). Furthermore, there were no comparative studies or tools to identify the most appropriate bioinformatic workflow for a specific cancer type. Five years on, to our knowledge, these issues remain largely unaddressed. Therefore, to improve the reproducibility and robustness of mutational signature calls, the implementation of standardized workflows is needed as well as computational tools to identify the influence of the variables on the analysis.

In this context, we used CRC as a model system to investigate how discrepancies due to different methodological approaches affect the determination of mutational signatures. We performed two complementary assessments: a) a mathematical evaluation, in which we calculated how accurately mutational signatures recapitulate the genetic landscape of cancer samples; b) a biological evaluation, in which we evaluated the identification of the MSI-MMRd/MSS-MMRp and the *POLE*-mutant status of CRC samples. Next, we conducted further validations of our results using three independent datasets, including a cohort of endometrial cancer patients, a cohort of lung cancer patients and a dataset of CRC PDOs.

We assessed how different bioinformatic tools, NGS workflows and reference catalogues influence the final outcome of the analysis. Our results show that the use of WGS data does not improve the ability to stratify biologically relevant CRC subtypes, highlighting the importance of appropriate experimental design for mutational signature analysis. In particular, we found that focusing on the coding regions for mutational signature fitting improved CRC stratification. Given the enrichment in coding sequences of the currently available NGS targeted panels, this finding becomes particularly relevant from a clinical perspective. Accordingly, we found that performing mutational signature fitting using large pan-cancer targeted gene panels for CRC subtypes stratification is technically effective, reliable and robust in terms of biological outcomes.

In addition, we found that the choice of algorithm led to statistically different results. In this regard, our study has limitations: for pragmatic reasons, we focused on five of the most used algorithms for performing mutational signature analysis; however, more than 30 different tools are currently present in literature (as of July, 2023). Furthermore, we selected a specific version of each of the five software and we cannot exclude that the results could slightly differ depending on the versions. Overall, we found that MP was the best choice in the CRC cell lines. In contrast, SPA was the preferred choice for CRC molecular stratification. The SA algorithm offers the best performance in cohort of samples with similar genetic features while it underperforms in case of sample cohorts with heterogenous genetic subtypes. To extend the benchmarking to a broader context, we further compared the tools with respect of aetiological and molecular tumor features. These included neoplasms with distinct DNA repair deficiencies, tumors associated with tobacco smoke and colibactin exposure such as samples from endometrial and lung cancer patients and a preclinical dataset of CRC PDOs. These extended analyses confirmed that the level of performance of MP exceeded that of other tools we evaluated.

The mutational signature reference is also relevant to the outcome of the analysis and should be chosen depending on the biological question. According to our results, reducing the number of signatures in the reference improved the stratification of CRC subtype (MSI-MMRd, MSS-MMRp, *POLE*-mutated), suggesting that TS or C2 repositories might be a better choice compared to C3 once ascertained that they contain all the signatures to be investigated in a particular experimental setting. Additionally, we have shown how the contribution of specific signatures vary depending on the mutational signature reference. This point becomes particularly relevant when evaluating the contribution of a unique signature linked to a specific aetiology, a condition already reported in literature in the case of MMR deficiency associated signatures, where specific signatures could be linked to different DNA repair mechanism deficiency [44, 45] or to specific genetic syndromes [46].

Our study indicated that the threshold for a reliable analysis depends on both the quantity and quality of mutations, considering artefacts and germline mutations. Relatedly, we observed a 50% reduction in artefacts associated signature levels when using only somatic variants from a matched analysis, suggesting the importance of matched normal or ‘metanormal’ samples to enhance mutational signature profiling.

Finally, to further improve the useability of our results and to help researchers to identify the most appropriate workflow in their setting, we developed CoMSCER, a bioinformatic tool which streamlines mutational signature analysis by evaluating the impact of multiple variables on the mutational signature profile. Specifically, by enabling users to quickly access parallel analyses using multiple algorithms and various mutational signature references, it can provide valuable insights into the reliability and consistency of the results. Moreover, CoMSCER provides information on the most appropriate reference which would reduce the frequency by which samples are excluded due to cosine similarity values. Finally, CoMSCER can evaluate how mutational signature profiling might vary across different genomic regions, whether coding or extragenic. Additionally, CoMSCER provides the functionality to filter regions using a metanormal, allowing to reduce the confounding effect of germline variants or systematic errors introduced during sequencing protocols.

All the data collection, software and workflows used in this study are freely available.

**Key Points**

- Distinct algorithms, references and genomic sizes produce statistically different results, highlighting the role of arbitrary choices in influencing mutational signature analyses.
- The study highlights a differential contribution of mutational signatures between coding and intergenic regions
- The minimum threshold of somatic variants required for reliable mutational signature assignment is investigated.
- Guidelines are proposed to guide researchers towards standard mutational signature analysis.
- The study presents CoMSCER, a bioinformatics tool that assists researchers in evaluating signature contributions across genomic regions and in identifying optimal workflows.

## Supplementary Material

FigureS1_bbae249

FigureS2_bbae249

FigureS3_bbae249

TableS1_bbae249

TableS2_bbae249

TableS3_bbae249

Figures_bbae249

## Author Biographies

**Paolo Battuello** is a PhD student in Complex Systems for Quantitative Biomedicine at University of Turin, Italy. His research interests include bioinformatics, oncology and genomics.

**Giorgio Corti** is a research fellow at the University of Turin, Italy. His research interests include bioinformatics and cancer genomics.

**Alice Bartolini** is a technologist in charge of the DNA_NGS Facility at the Candiolo Cancer Institute, FPO - IRCCS, Candiolo, Italy. Her research interests include NGS approaches applied to preclinical and translational cancer research.

**Annalisa Lorenzato** has a permanent position as research technician at University of Turin, Turin, Italy. Her research interests include molecular biology, cancer genetics, 2D and 3D cell culture models.

**Alberto Sogari** is a postdoctoral fellow at the Department of Oncology of the University of Torino. His research interests include resistance to anticancer therapy and DNA repair in cancer cells.

**Mariangela Russo** is an Assistant Professor at Dept of Oncology, University of Turin, Italy. Her research interests include molecular genomics, cancer genetics, tumor evolution and resistance to targeted therapy.

**Federica Di Nicolantonio** is an associate professor of Biochemistry at the Department of Oncology, University of Turin, Italy and Group Leader of Laboratory of Cancer Epigenetics at Candiolo Cancer Institute – FPO, IRCCS Torino, Italy. Her research interests include biochemistry, genomics, cancer genetics and oncology.

**Alberto Bardelli** is a full professor of histology at the Department of Oncology, University of Turin, Italy, Scientific Director and group leader at IFOM, the AIRC Institute of Molecular Oncology, Milan Italy. His research interests include molecular genomics, cancer genetics and precision medicine in oncology.

**Giovanni Crisafulli** is a computational scientist - bioinformatician - at IFOM, the AIRC Institute of Molecular Oncology, Milan Italy. His research interests include bioinformatics, genomics, cancer genetics, oncology and precision medicine.
