# Supplementary figures and images for: Mutational signatures of colorectal cancers according to distinct computational workflows
Source: Brief Bioinform. 2024 May 23;25(4):bbae249. doi: 10.1093/bib/bbae249 (PMC11116831; doi:10.1093/bib/bbae249)

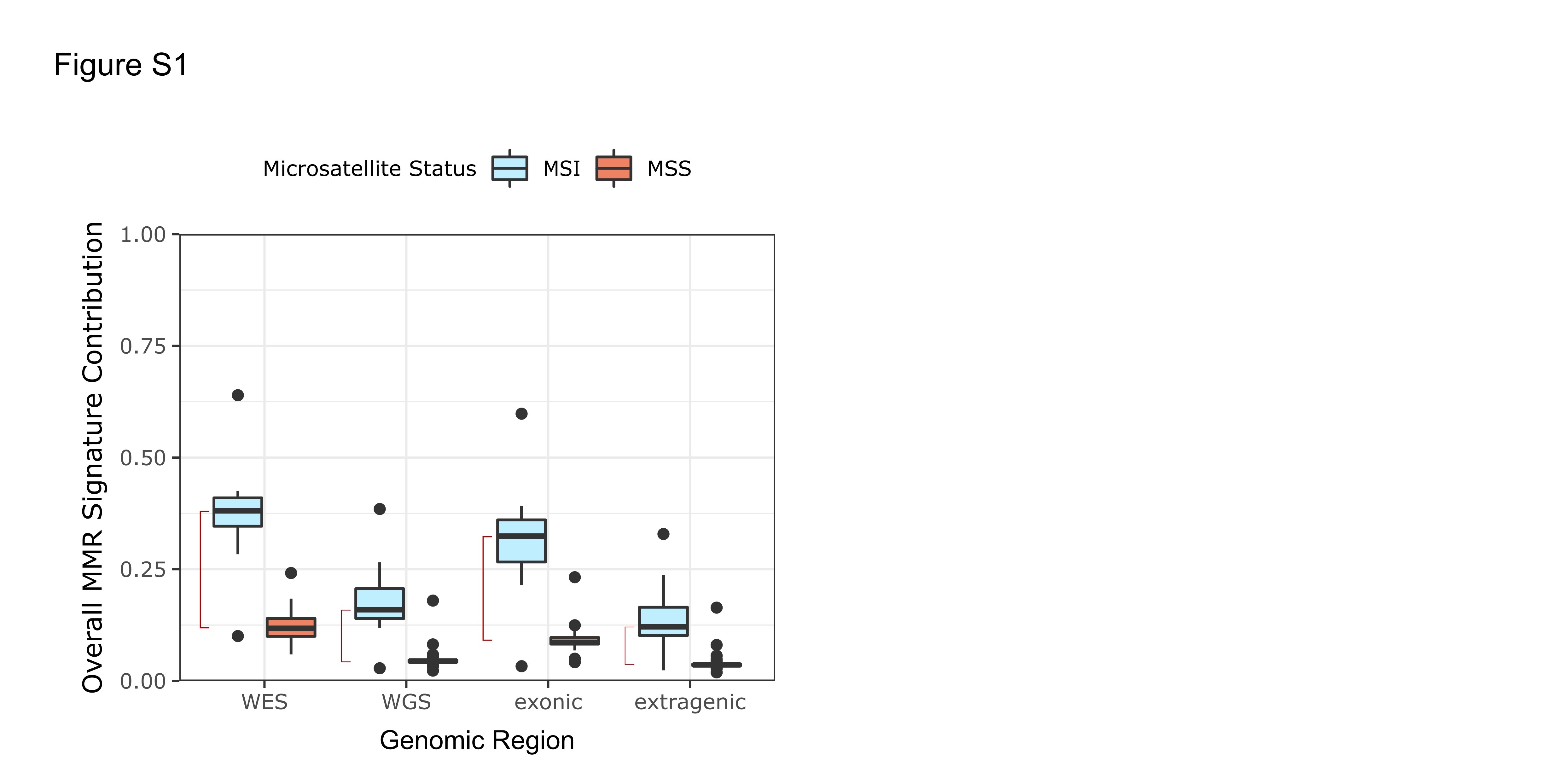

Supplement: FigureS1_bbae249 [file figures1_bbae249.jpeg]

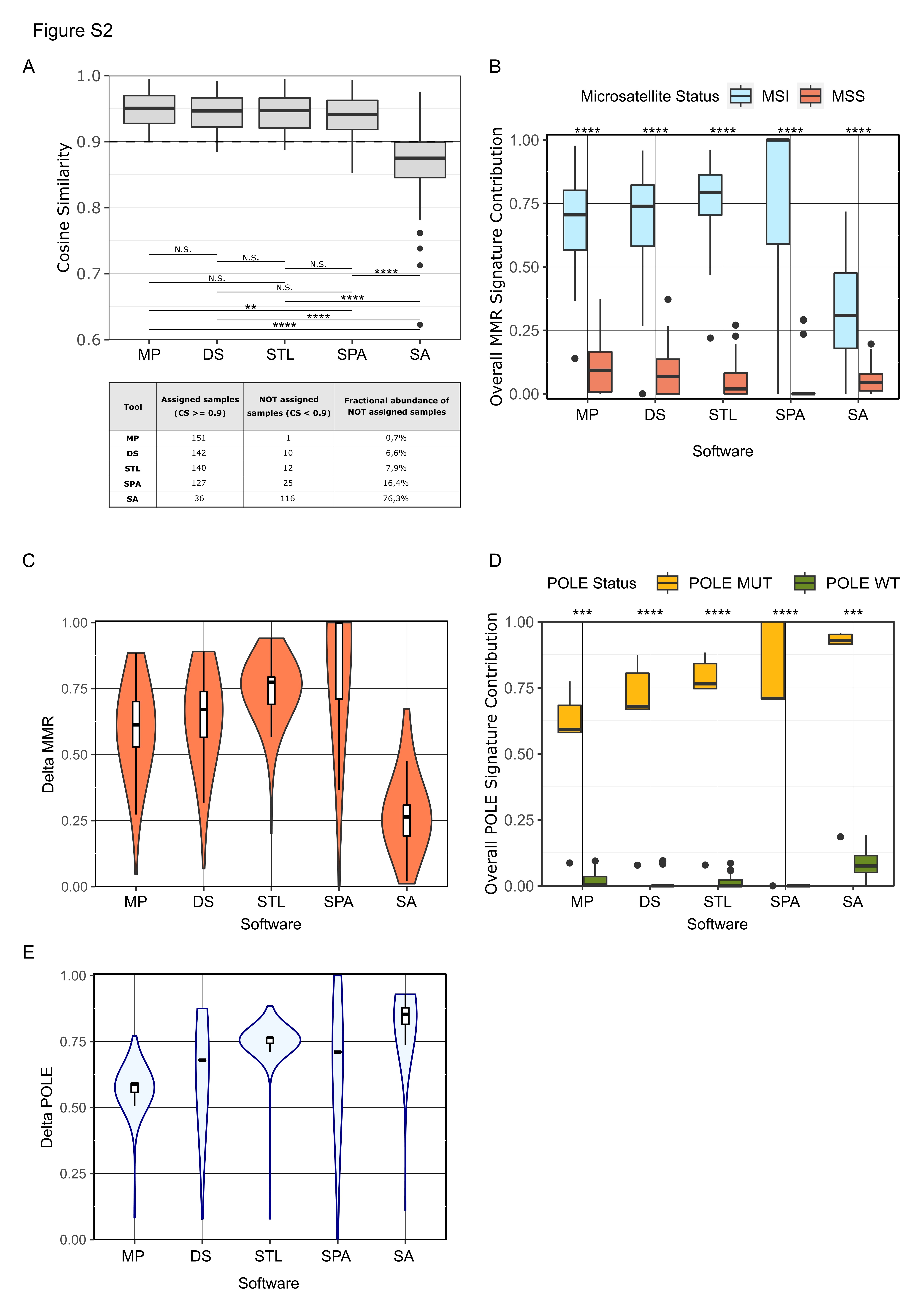

Supplement: FigureS2_bbae249 [file figures2_bbae249.jpeg]

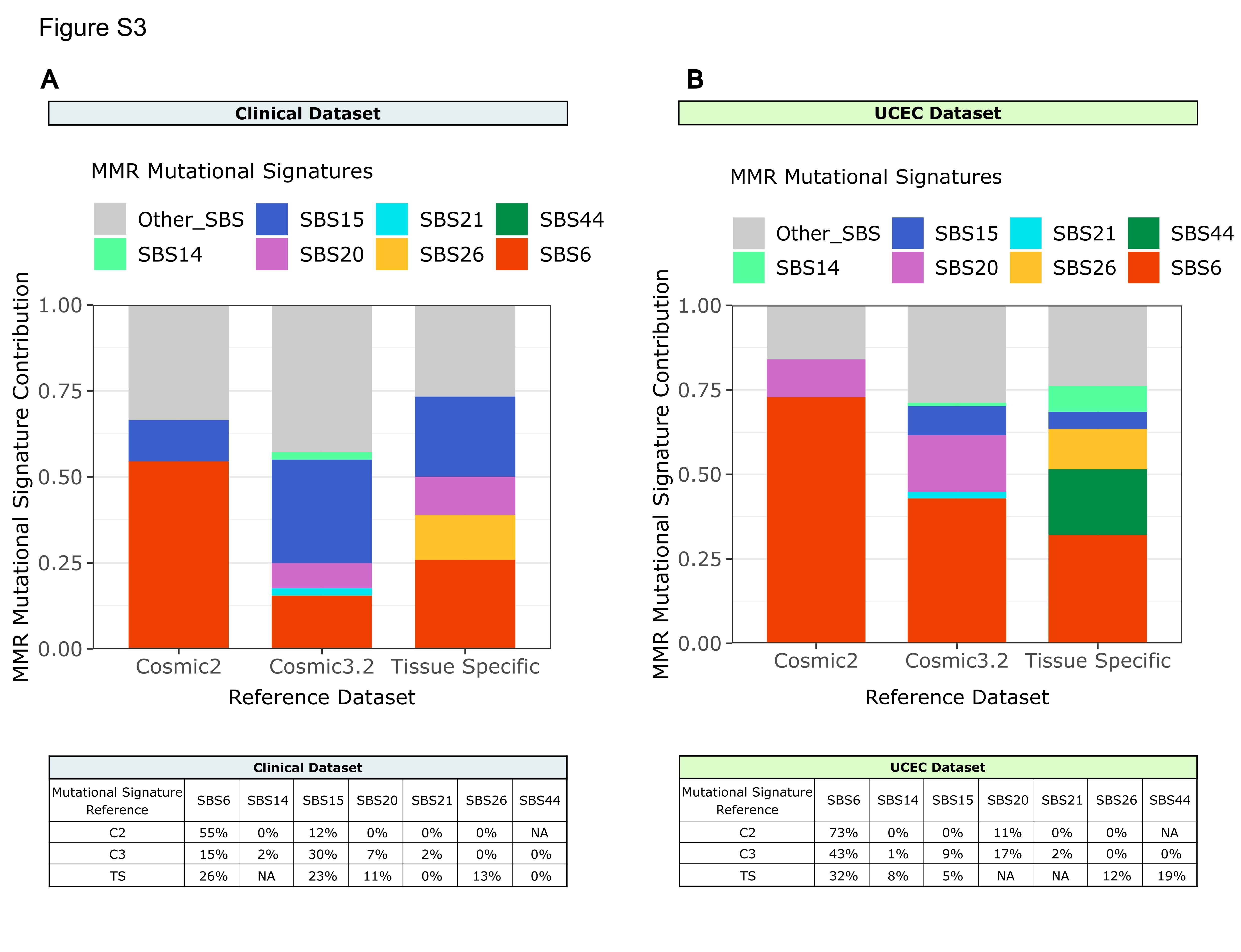

Supplement: FigureS3_bbae249 [file figures3_bbae249.jpeg]
