# Supplementary material for: Mutational signatures of colorectal cancers according to distinct computational workflows
Source: Brief Bioinform. 2024 May 23;25(4):bbae249. doi: 10.1093/bib/bbae249 (PMC11116831; doi:10.1093/bib/bbae249)
Supplement: Figures_bbae249 [file figures_bbae249.docx]

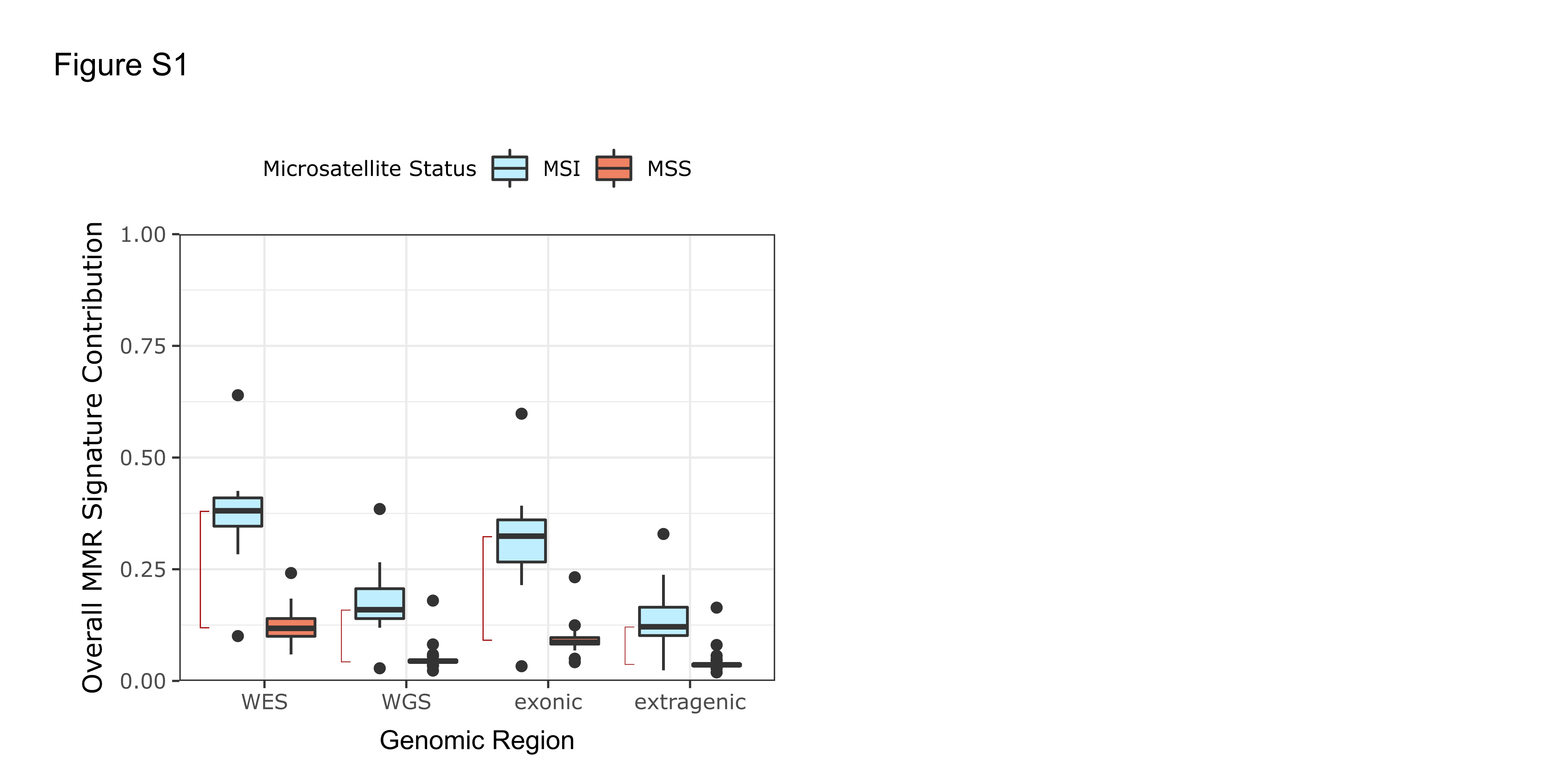


**Figure S1**. *Evaluation of mutational signature contribution from coding and extragenic regions.* (A) Overall contribution of MMRd-associated signatures in MSI-MMRd and MSS-MMRp CRC cell lines using WES, WGS and from exonic and extragenic regions extracted from WGS data. *MMR Mismatch Repair, WES Whole Exome Sequencing, WGS Whole Genome Sequencing*


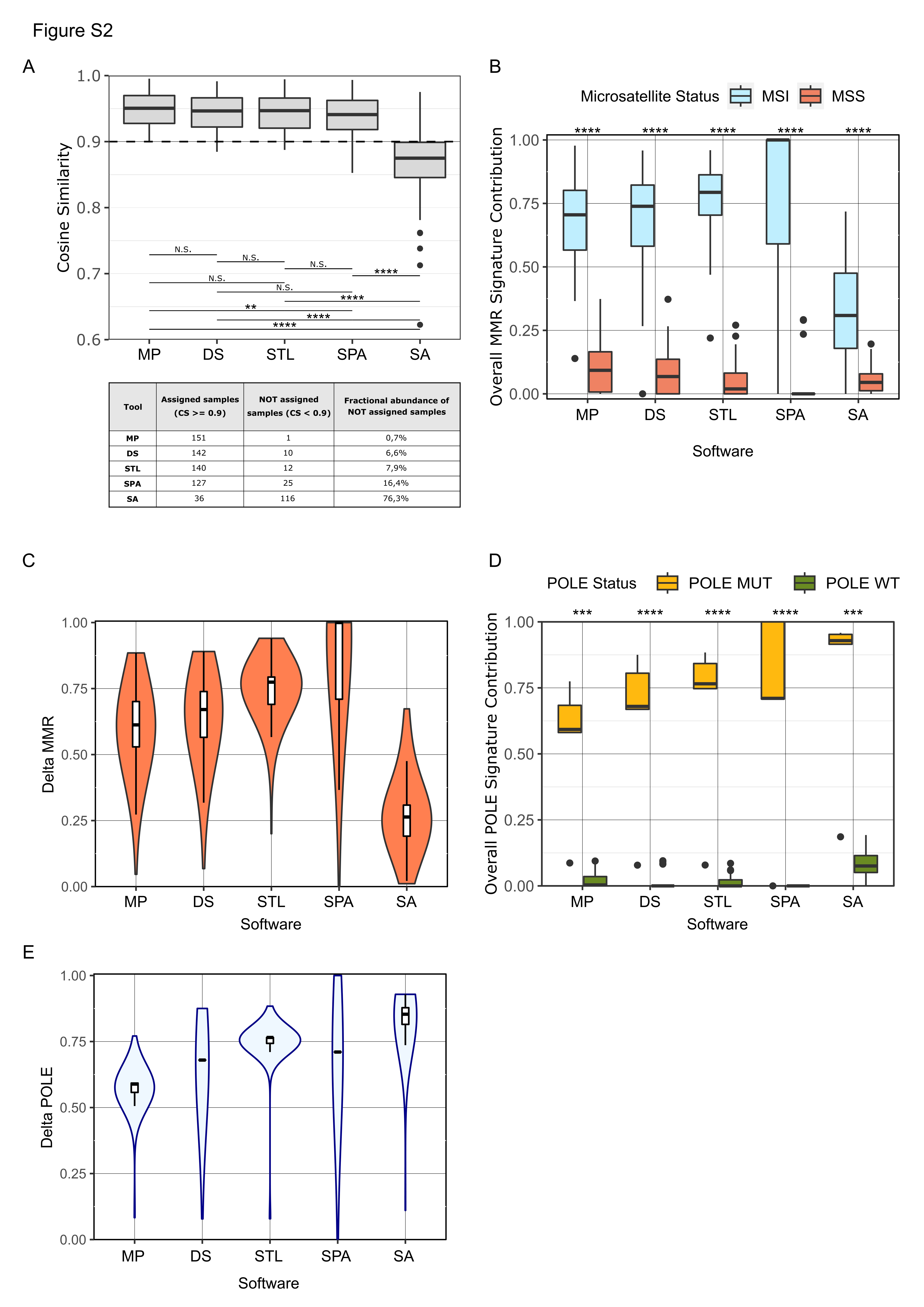


**Figure S2.** *Impact of algorithm choice on mutational signatures analysis in the clinical dataset*. (A) Top panel: distribution of cosine similarity values obtained with *MutationalPatterns, deconstructSigs, signature-tools.lib, SigProfilerAssignment and signatureanalyzer* in the preclinical datasets. Lower panel: details of the number of samples that, upon mutational signature analysis, reach cosine similarity value greater or equal than 0,9 for each tool. (B) Overall contribution of MMRd associated signatures in MSI-MMRd and MSS-MMRp samples using five different tools. (C) Distribution of delta MMRd values using multiple bioinformatic tools: *MP MutationalPatterns, DS deconstructSigs, STL signature-tools.lib, SPA SigProfilerAssignment, SA signatureanalyzer*. (D) Overall contribution of POLE associated mutational signatures according to the different tools. (E) Distribution of delta POLE values using the five tools.*, n.s. not significant*.


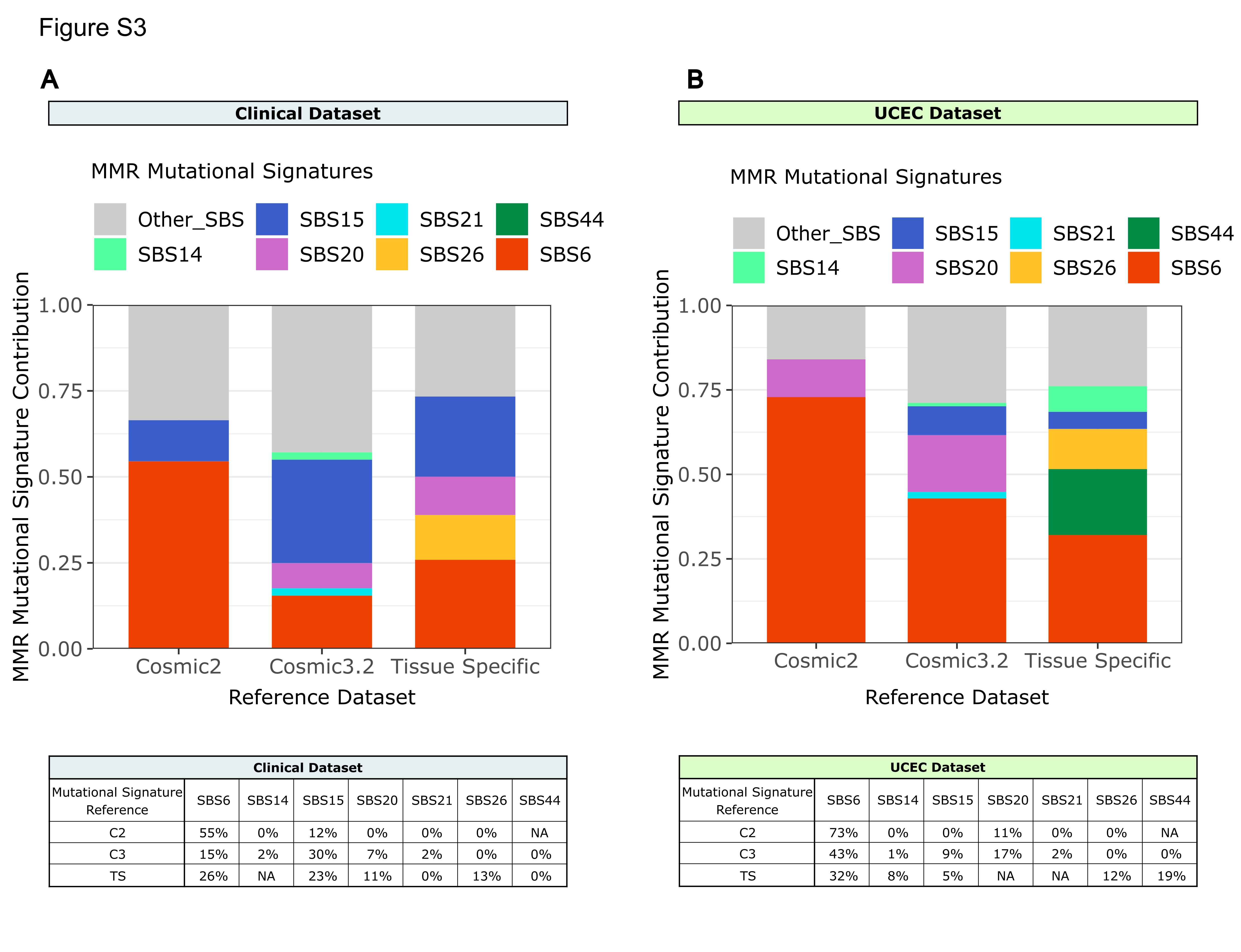


**Figure S3.** *Impact of the reference on mutational signatures analysis.* (A) Normalised contribution of individual MMR-associated signatures in the MSI-MMRd cohort of the CRC clinical dataset. (B) Normalised contribution of single MMR-associated signatures in the MSI-MMRd cohort of the endometrial cancer dataset. *MMR Mismatch Repair.*
